# Impact of a Mobile Phone Intervention to Reduce Sedentary Behavior in a Community Sample of Adults: A Quasi-Experimental Evaluation

**DOI:** 10.2196/jmir.5137

**Published:** 2016-01-25

**Authors:** Darla E Kendzor, Kerem Shuval, Kelley Pettee Gabriel, Michael S Businelle, Ping Ma, Robin R High, Erica L Cuate, Insiya B Poonawalla, Debra M Rios, Wendy Demark-Wahnefried, Michael D Swartz, David W Wetter

**Affiliations:** ^1^ Department of Family and Preventive Medicine University of Oklahoma Health Sciences Center Oklahoma City, OK United States; ^2^ Oklahoma Tobacco Research Center Stephenson Cancer Center Oklahoma City, OK United States; ^3^ Intramural Research Department Economic and Health Policy Research Program American Cancer Society Atlanta, GA United States; ^4^ School of Public Health Department of Epidemiology, Human Genetics and Environmental Sciences The University of Texas Health Science Center Austin, TX United States; ^5^ Department of Clinical Sciences The University of Texas Southwestern Medical Center Dallas, TX United States; ^6^ College of Public Health Department of Biostatistics University of Nebraska Medical Center Omaha, NE United States; ^7^ School of Public Health University of Texas Health Science Center Dallas, TX United States; ^8^ Department of Nutrition Sciences The University of Alabama at Birmingham Birmingham, AL United States; ^9^ School of Public Health Department of Biostatistics The University of Texas Health Science Center Houston, TX United States; ^10^ Department of Psychology Rice University Houston, TX United States

**Keywords:** sedentary lifestyle, mobile phone, African Americans, physical activity

## Abstract

**Background:**

Greater time spent sedentary is linked with increased risk of breast, colorectal, ovarian, endometrial, and prostate cancers. Given steadily increasing rates of mobile phone ownership, mobile phone interventions may have the potential to broadly influence sedentary behavior across settings.

**Objective:**

The purpose of this study was to examine the short-term impact of a mobile phone intervention that targeted sedentary time in a diverse community sample.

**Methods:**

Adults participated in a quasi-experimental evaluation of a mobile phone intervention designed to reduce sedentary time through prompts to interrupt periods of sitting. Participants carried mobile phones and wore accelerometers for 7 consecutive days. Intervention participants additionally received mobile phone prompts during self-reported sitting and information about the negative health impact of prolonged sedentariness. The study was conducted from December 2012 to November 2013 in Dallas, Texas. Linear mixed model regression analyses were conducted to evaluate the influence of the intervention on daily accelerometer-determined estimates of sedentary and active time.

**Results:**

Participants (N=215) were predominantly female (67.9%, 146/215) and nonwhite (black: 50.7%, 109/215; Latino: 12.1%, 26/215; other: 5.6%, 12/215). Analyses revealed that participants who received the mobile phone intervention had significantly fewer daily minutes of sedentary time (B=–22.09, *P*=.045) and more daily active minutes (B=23.01, *P*=.04) than control participants.

**Conclusions:**

A simple mobile phone intervention was associated with engaging in less sedentary time and more physical activity. Findings underscore the potential impact of mobile phone interventions to positively influence sedentary behavior and physical activity.

## Introduction

Sedentary behavior has been defined as any activity that requires an energy expenditure no greater than 1.5 metabolic equivalents (METs) that is performed while sitting or reclining [1]. Exploring the health effects of sedentary behavior, independent of physical activity, has been a relatively new scientific pursuit, with a proliferation of studies published in the past decade. Evidence to date suggests that prolonged sedentary time is associated with increased risk for a variety of adverse health outcomes [2-12], including cancers of the breast, colon/rectum, ovaries, endometrium, and prostate [11-14]. In addition, greater sedentary time among adults is associated with weight gain, higher body mass index (BMI), and obesity [15-20], which is a known risk factor for cancer [21]. Nevertheless, adults in the United States are excessively sedentary with an average of approximately 8 hours per day spent sedentary during waking hours [22]. This high level of sedentary time has been observed in both men and women and across several racial/ethnic groups [22]. Emerging research has indicated that inactive-to-active transitions (henceforth “sedentary breaks”) are linked with lower waist circumference, BMI, triglycerides, 2-hour glucose levels, and blood pressure [23-25]. Thus, interventions designed to reduce total sedentary behavior by interrupting prolonged sedentary bouts may have a substantial impact on health.

Despite the accumulating evidence supporting the deleterious health effects of prolonged sedentariness, few interventions have specifically focused on decreasing and interrupting sedentary time. Because engaging in habitual physical activity often requires significant effort and planning, it seems likely that modifying sedentary behavior through periodic interruptions during waking hours may be more achievable and sustainable over time. It is possible that modifying sedentary behavior represents a less complex behavior change, especially for inactive individuals [26,27]. Notably, Bond et al [28] recently reported promising initial findings demonstrating that a mobile phone intervention was associated with reductions in sedentary time among overweight/obese adults. The findings of a recent meta-analysis provide initial evidence that sedentary behavior interventions significantly reduce sedentary time [29], although most intervention studies have focused specifically on reducing occupational sitting time or screen time rather than total daily sitting time.

Recent research indicates that 64% of US adults owned mobile phones in 2015 [30], suggesting that mobile phone interventions have the potential to broadly influence sedentary behavior among adults across diverse settings. Thus, the purpose of the current study was to characterize the impact of a mobile phone-based sedentary behavior intervention that incorporated education, self-monitoring, and prompting in a community sample of adults. It was hypothesized that participants who received mobile phone prompts to decrease sedentary time would have significantly less sedentary time, more active time, and more sedentary breaks than those who did not receive mobile phone prompts over a 7-day period. In addition, it was anticipated that participants who received prompts to increase activity would be more acutely active following self-reported sitting than those who did not receive prompts.

## Methods

### Participants

A total of 248 adults were recruited from the Dallas metropolitan area through flyers posted on the University of Texas Southwestern campus (Dallas, TX), local advertising circulars, and word of mouth. Individuals were eligible to participate in the study if they were at least 18 years of age, possessed a valid home address and a functioning telephone number, and demonstrated greater than 6th grade English literacy level on the Rapid Estimate of Adult Literacy in Medicine (REALM) [31,32]. Of those screened, 10 were excluded because they were not able to demonstrate the minimum reading level, leaving a total study sample of 238 participants. Data collection began in December 2012 and concluded in November 2013.

### Measures

#### Socioeconomic Status/Demographic Variables

Race/ethnicity, sex, age (in years), and educational attainment were assessed.

#### Body Mass Index

Participant’s BMI was calculated based on objective measurements of height and weight using the standard formula (kg/m^2^).

#### Smoking

Expired carbon monoxide (CO) levels were measured with a portable Vitalograph ecolyzer, which provided an objective indicator of current smoking status and level of smoking. CO levels of ≥8-10 parts per million (ppm) suggest recent cigarette smoking with a sensitivity and specificity of approximately 90% [33].

#### Sedentary Behavior and Physical Activity

Sedentary time at baseline was based on responses to 2 items from the International Physical Activity Questionnaire (IPAQ). The IPAQ assessed usual time spent sitting on a weekday and on a weekend day during the past week [34]. Weekday estimates were multiplied by 5, weekend day estimates were multiplied by 2, and the resulting values were summed and divided by 7 to calculate the mean daily time spent sitting during the past week.

Physical activity and sedentary time were directly assessed using Actigraph GT3X (Pensacola, FL) triaxial accelerometers. Accelerometers were initialized via ActiLife6 software to begin data collection at midnight on the day of the baseline visit. Participants were instructed to wear the accelerometer on the waist and in line with their right hip, secured using an elastic belt during all waking hours for 7 consecutive days. Participants were asked to remove the accelerometer when sleeping, bathing or showering, and during all water activities. After the 7-day data collection period was completed, the monitors were returned at a second in-person visit and data were downloaded via ActiLife6 software. Before data reduction and processing, the downloaded data files were reintegrated and expressed as 60-second epochs. A 60-second epoch was used for consistency with previous research in nationally representative samples [22]. Research has shown that associations of activity estimates with key outcomes are not markedly different when shorter or longer epochs are used [35].

During the data reduction and processing stage, data were screened for periods of nonwear using established methods [36,37]. Nonwear periods were removed from further analysis. Total activity counts per day were calculated using summed daily counts detected over wear periods. Minutes spent in sedentary activity, as well as light and moderate lifestyle intensity activity were estimated using Matthews cut-points for all days with 10 hours or more per wear time [38]. Specifically, activity count ranges were 0 to 99 counts per minute for sedentary activity, 100 to 759 counts per minute for light-intensity activity, and 760 to 1951 counts per minute for moderate lifestyle intensity activity. An estimate reflecting total time spent active was also created using accumulated time ≥100 counts per minute. Sedentary breaks were defined as any period of sedentary time (ie, <100 counts/minute) that was immediately followed by a minute or more of active time (≥100 counts) [24] and sedentary breaks were summed across all waking hours.

#### Mobile Phone Assessments

All participants were provided with an Android mobile phone on which they were prompted to complete daily diary assessments and random assessments of health behavior and psychosocial variables (as part of a parent study; see Procedure section) over a 7-day observation period. Participants completed daily diary assessments once daily, 30 minutes after their self-reported usual wake time. In addition, participants were randomly prompted to complete assessments 4 times per day during self-reported waking hours. Participants were required to complete mobile phone assessments within 15 minutes, although they were allowed to postpone assessments by 5 minutes for a total of 3 times. Of relevance to the current analyses, participants responded to the following daily diary and random assessment items, respectively: (1) “How many hours did you spend sitting yesterday?” and (2) “What were you doing right before your phone rang/vibrated?” Response options included sitting, talking, standing, walking/exercising, sleeping/resting, or other.

### Procedure

**Figure 1:**
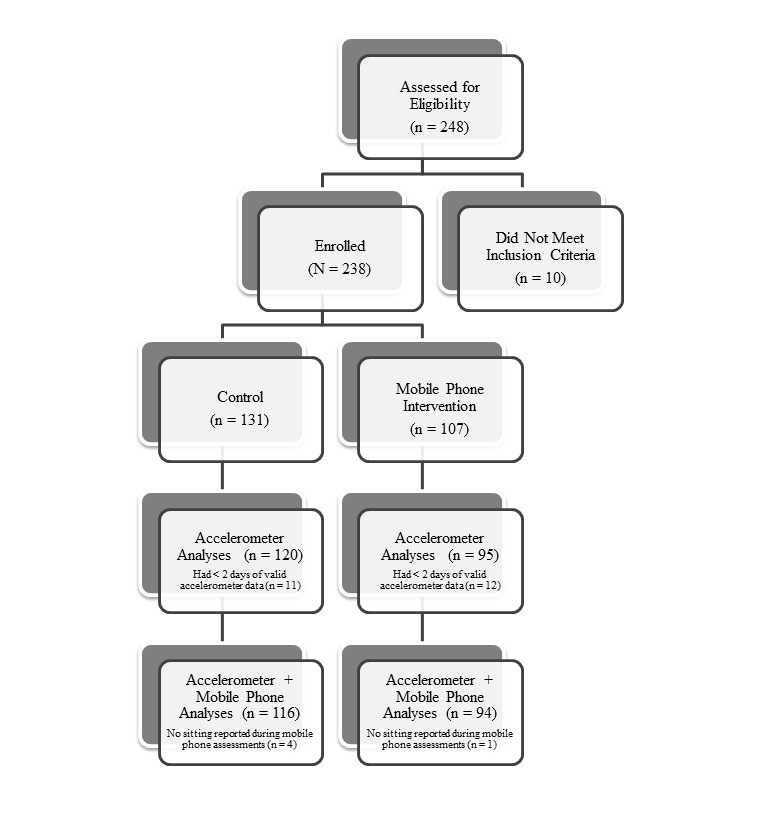
Participant flow for nonrandomized intervention trial.

The current study was approved by the Institutional Review Boards of the University of Texas Southwestern Medical Center and the University of Texas Houston Health Science Center. The sedentary behavior intervention described here was a post hoc addition to an observational prospective 7-day study. The parent study was designed to characterize proximal predictors of health behavior using mobile phone–based ecological momentary assessment. Thus, the current study had a quasi-experimental (nonrandomized) design whereby the first 131 consecutive participants who did not receive the intervention served as the control group. Control participants completed mobile phone assessments and wore an accelerometer to measure sedentary and active time over 7 consecutive days. The subsequent 107 participants who enrolled in the study additionally received the sedentary behavior intervention on the mobile phone. Participant recruitment and group allocation are depicted in Figure 1.

Potential participants were provided with the details of the study over the telephone and their interest in participating was assessed. Interested individuals were briefly screened by phone for eligibility and those eligible were scheduled to attend the initial study visit. The details of the study were reviewed at the first visit and informed consent was obtained from all participants. Reading level was assessed and participants who were unable to demonstrate greater than 6th grade reading level on the REALM were excluded from the study and compensated for their time with a US $20 gift card and a parking token.

Eligible participants completed study questionnaires on laptop computers. Height, weight, and CO were measured by trained staff. Participants were provided with a mobile phone and an accelerometer, instructed in their use, and asked to wear/carry the devices for 7 days. Participants received a US $50 gift card and a parking token for the completion of the baseline visit. Participants returned for a final visit and received up to US $80 in gift card compensation depending on the percentage of mobile phone assessments completed. A mobile phone assessment completion rate of 80% and the return of study mobile phones and accelerometers were required to earn the maximum compensation.

### Intervention

Intervention information and prompts used in the current study were based on previously developed messages and materials [39,40]. The intervention group received a 1-page printed handout at the first visit describing the health-related importance of limiting sedentary time and increasing activity. The handout included suggestions about ways to reduce sedentary time and increase light-intensity activity throughout the day (eg, by moving around in the office). In addition, a daily message used in previous research [39] appeared on each participant’s mobile phone at the end of daily diary assessment for 7 days: “Remember to STAND UP, SIT LESS, and MOVE MORE today!”

During the 7-day intervention period, participants who reported more than 2 hours of sitting during the previous day via the morning daily diary assessment received the following message: “Medical research has shown that long periods of uninterrupted sitting increase the likelihood of several health problems, including obesity and Type 2 diabetes. Make an effort to Stand Up more, Sit Less, and Move About more. This can be achieved by taking frequent standing and walking breaks (at least one break for every half hour of sitting), standing up when talking on the phone (at work or home), checking emails, etc, and replacing blocks of sitting time with standing time, such as doing household chores while watching TV.” This message was adapted from previous research [39].

Participants who reported that they were sitting during any random mobile phone assessment received the following message: “Medical research has shown that long periods of uninterrupted sitting increase the likelihood of several health problems, including obesity and Type 2 diabetes. Please consider standing up *now* and moving about for 5 minutes. Make an effort to improve your health by standing up and moving around your home or office every half hour during periods of sitting.” Note that both intervention and control participants completed mobile phone, questionnaire, anthropometric, and accelerometer assessments. However, only intervention participants received the education and mobile phone messages prompts.

### Statistical Analyses

A series of linear mixed model (LMM) regression analyses were conducted to evaluate the influence of the sedentary behavior intervention (relative to the control group) on accelerometer-measured daily active minutes as well as daily minutes of sedentary, light, and moderate lifestyle activity over 7 days. Model 1 adjusted for daily minutes of wear time and study day. Model 2 adjusted for race (white vs nonwhite), education (≤high school vs >high school), CO level (ppm), age (in years), daily minutes of wear time, and study day (day 1-7). Total number of daily sedentary breaks was also examined as an outcome, with daily minutes of sedentary time additionally included in the models. Participants who did not have at least 2 days of accelerometer wear time of at least 10 hours per day were excluded from these analyses (n=23), leaving an analytic sample of 215 participants.

Additional analyses were conducted to compare active minutes and accelerometer counts between the groups during the 10 minutes following random mobile phone assessments where sitting was reported. A total of 5 participants did not endorse sitting during any random assessments and, therefore, the sample size was reduced to 210 participants in these analyses only. Model 1 adjusted for total random assessments completed, daily minutes of wear time, time of random assessments when sitting was endorsed, and study day. Model 2 adjusted for race, education, CO level (smoking), age, daily minutes of wear time, time of random assessments when sitting was endorsed, and study day.

## Results

### Participant Characteristics

Participants (N=215) were predominantly female (67.9%, 146/215) and nonwhite (black: 50.7%, 109/215; Latino: 12.1%, 26/215; other: 5.6%, 12/215). See Table 1 for participant characteristics overall and by intervention group. Participants in the intervention group (n=95) were older, had higher CO levels, were more likely to be nonwhite, and were less likely to have completed greater than a high school education than those in the control group (n=120). Participants did not differ significantly by intervention group on self-reported mean daily sitting at baseline (mean 6.90 hours per day, SD 3.71), mean daily accelerometer wear time during the study (mean 843.63 minutes per day, SD 99.38), or days of accelerometer wear (mean 5.87 days, SD 1.45). Overall, participants completed 87.2% (mean 24.42, SD 4.15) of 28 possible random assessments via mobile phone over the 7-day study period, although control group participants had a slightly higher completion rate than those in the intervention group (mean 24.93, SD 3.35 vs mean 23.80, SD 4.90 completed assessments, *P*=.049).

Descriptive analyses of accelerometer estimates overall and by intervention group are presented in Table 2. Note that unadjusted comparisons indicate that intervention participants had significantly fewer daily accelerometer-measured sedentary minutes, spent less of their total accelerometer wear time in sedentary activity, and spent more of their daily accelerometer wear time active and engaged in light-intensity activity. Active minutes and total accelerometer counts in the 10-minute postprompt period were also greater in the intervention group.

**Table 1:** Baseline participant characteristics (N=215).

Participant characteristics	Total sample (N=215)	Intervention group (n=95)	Control group (n=120)	*P* ^a^
Race (nonwhite), n (%)	147 (68.4)	77 (81)	70 (58.3)	<.001
Gender (female), n (%)	146 (67.9)	66 (70)	80 (66.7)	.66
Age (years), mean (SD)	43.90 (12.85)	46.75 (11)	41.65 (13.62)	.004
Education (>high school), n (%)	160 (74.4)	55 (58)	105 (87.5)	<.001
BMI (kg/m^2^), mean (SD)	30.72 (7.81)	31.61 (8)	30.02 (7.90)	.14
CO level (ppm), mean (SD)	6.49 (10.18)	9.2 (11)	4.35 (8.46)	<.001

^a^Variables that were found to differ significantly between the intervention and control groups were included as covariates in adjusted analyses.

### Intervention

The LMM regression analyses indicated that participants who received the sedentary behavior intervention had significantly fewer accelerometer-measured daily minutes of sedentary time and more daily active minutes over the 7-day study period than participants who did not receive the intervention in adjusted models 1 and 2 (see Table 3). Analyses indicated that those included in the mobile phone intervention group engaged in significantly more minutes of light-intensity activity than control group participants in model 1 only. Daily minutes of moderate lifestyle intensity activity and total daily sedentary breaks did not differ significantly between groups in either model. Additional analyses indicated that intervention participants had significantly more active minutes (B=0.33, *P*=.01) and accelerometer counts (B=350.67, *P=*.01) than control participants in the 10 minutes following random assessments where sitting was endorsed after adjustment for total random assessments completed, study day, time of random assessment when sitting, and daily minutes of accelerometer wear time. However, when race, smoking (CO level), age, and education were added to the model, results were no longer significant for active minutes (B=.18, *P*=.21) or accelerometer counts (B=283.31, *P*=.06).

**Table 2:** Daily accelerometer estimates across valid wear days overall and by intervention group (N=215).

Accelerometer variables^a^	Total sample (N*=*215)	Intervention group (n=95)	Control group (n=120)	*P*
Mean daily wear time (minutes/day), mean (SD)	843.63 (99.38)	829.96 (96.28)	854.46 (100.86)	.07
Total days of observation (out of 7 possible), mean (SD)	5.87 (1.45)	5.74 (1.52)	5.98 (1.39)	.23
Sedentary, daily minutes, mean (SD)	531.64 (100.96)	507.20 (101.01)	550.99 (97.04)	.001
Sedentary, % of daily wear time, mean (SD)	62.92 (8.89)	61.08 (9.72)	64.38 (7.91)	.007
Active, daily minutes, mean (SD)	310.94 (80.59)	322.37 (88.01)	301.88 (73.30)	.06
Active, % of daily wear time, mean (SD)	36.96 (8.88)	38.87 (9.71)	35.44 (7.88)	.005
Light intensity, daily minutes, mean (SD)	215.12 (55.52)	222.83 (56.50)	209.01 (54.19)	.07
Light intensity, % of daily wear time, mean (SD)	25.54 (5.95)	26.87 (5.93)	24.49 (5.78)	.003
Moderate lifestyle intensity, daily minutes, mean (SD)	71.54 (31.07)	74.80 (35.90)	68.96 (26.51)	.17
Moderate lifestyle intensity, % of daily wear time, mean (SD)	8.53 (3.70)	9.03 (4.27)	8.14 (3.13)	.08
Inactive-to-active transitions, daily total, mean (SD)	94.17 (17.03)	94.53 (16.25)	93.88 (17.70)	.78
Total active minutes (10 minutes postprompt), mean (SD)^b^	2.44 (1.02)	2.59 (1.15)	2.32 (0.88)	.05
Total accelerometer counts (10 minutes postprompt), mean (SD)^b^	1782.07 (1096.64)	1970.61 (1322.76)	1629.29 (847.40)	.03

^a^Accelerometer estimates were defined as follows: sedentary activity was defined as less than 100 counts per minute, active time was defined as 100 or more counts per minute, light-intensity activity was defined as 100-759 counts per minute, and moderate lifestyle intensity activity was defined as 760-1951 counts per minute. An inactive-active transition (ie, sedentary break) was defined as a transition from less than 100 counts to 100 or more counts/minute.

^b^ Sample size slightly reduced (N=210) because 5 participants had no reports of sitting during random mobile phone assessments.

**Table 3:** Effects of a mobile phone intervention on accelerometer-measured activity over 7 days (N=215).^a^

Accelerometer variables^b^	Model 1^c^		Model 2^d^	
	Unstandardized coefficient	*P*	Unstandardized coefficient	*P*
Sedentary, daily minutes	–27.33	.007	-22.09	.045
Active, daily minutes	28.52	.005	23.01	.04
Light intensity, daily minutes	18.94	.005	11.73	.10
Moderate lifestyle intensity, daily minutes	7.85	.06	7.14	.12
Inactive-to-active transitions, daily total^e^	3.06	.09	1.15	.56

^a^In the analyses, no intervention=0 and intervention=1.

^b^ Accelerometer estimates were defined as follows: sedentary activity was defined as <100 counts per minute, active time was defined as ≥100 counts per minute, light-intensity activity was defined as 100-759 counts per minute, and moderate lifestyle intensity activity was defined as 760-1951 counts per minute. An inactive-active transition (ie, sedentary break) was defined as a transition from <100 counts to ≥100 counts/minute.

^c^ Adjusted for daily minutes of accelerometer wear time and time/day.

^d^ Adjusted for race, education, CO level, age, daily minutes of accelerometer wear time, and time/day.

^e^ Daily minutes of sedentary time was additionally included in the models.

## Discussion

The current study was among the first to evaluate a mobile phone intervention aimed at reducing sedentary behavior among adults of diverse racial/ethnic backgrounds. Findings indicated that intervention participants had significantly fewer minutes of daily sedentary time and more daily minutes of active time than controls over the 7-day study period. Daily minutes of light-intensity activity was significantly higher among intervention participants than those assigned to the control group in the partially adjusted model, although differences did not reach statistical significance in the fully adjusted model. Additionally, supplementary analyses indicated that activity was greater in the 10 minutes following self-reported sitting among intervention participants who received activity prompts than among control participants who did not receive prompts, although differences did not reach significance in the fully adjusted models. Overall, simple mobile phone prompts appear to be a promising strategy for reducing sedentary behavior and increasing activity, although adequately powered and well-designed studies will be needed to confirm these preliminary findings.

Intervention participants evidenced 3% less objectively measured sedentary time (of total accelerometer wear time) than control participants. To illustrate, 3% of 14 hours of mean wear time equals 25 minutes of time spent engaged in activity rather than in sedentary behavior. Differences in sedentary time noted in the current study are similar to the reductions reported with other types of sedentary intervention strategies [41,42]. Bond et al [28] specifically evaluated a mobile phone-based intervention using a within-subjects design and showed 3.3% to 5.9% decreases in sedentary time and 1.9% to 3.9% increases in light physical activity across 3 variations of the intervention. Although it is not certain whether these reductions in sedentary time (and increases in activity) have a significant impact on health, it is notable that differences in the current study were found using a very simple intervention which entailed (1) a printed handout, (2) a mobile phone reminder to “stand up, sit less, and move more” each morning, and (3) mobile phone prompts triggered by self-reported sitting several times daily. It is unclear why the intervention did not seem to impact sedentary breaks, although one possible explanation may be that the intervention was not intensive enough. Mobile phone interventions that are more intensive and those that use prompts based on real-time activity monitoring may have a greater impact on behavior.

Utilizing mobile phones to modify sedentary behavior is advantageous because phones can be used in most settings where individuals are sedentary, such as in the home or workplace [43]. In addition, rates of mobile phone ownership are steadily increasing, with the majority of US adults reporting that they owned a mobile phone in 2015 [30]. Although mobile phone interventions have the potential to broadly influence lifestyle behaviors, they have not been widely employed to modify sedentary behavior among adults. To date, most sedentary behavior interventions have focused primarily on reducing occupational sitting through the introduction of sit-stand desks and encouragement to use them throughout the day [39,44]. Although the workplace is an important place to target sedentary behavior, mobile phones offer the potential to influence sedentary behavior across settings where sedentary behavior is likely to take place. Plausibly, substantial reductions in sedentary behavior may reduce the likelihood of developing cancer and other diseases.

Study findings complement the findings of Bond et al [28], who tested 3 versions of a mobile phone intervention that provided feedback about time spent in objectively measured sedentary behavior and prompted activity breaks following periods of continuous sedentariness in a sample of overweight/obese adults who were predominantly white and female. Notably, all versions of the mobile phone intervention were associated with within-subjects decreases in sedentary time, although mobile phone prompts to engage in 3 minutes of activity after 30 minutes of sedentary time were associated with greater reductions in sedentary time than prompts to engage in 12 minutes of activity after 120 minutes of sedentary time. Future research will be needed to determine whether mobile phone interventions have a sustained impact on sedentary behavior and to determine the optimal scheduling of prompts in longer-term interventions.

This study has notable strengths and limitations. Strengths include the application of novel technology that is scalable and has the potential to modify sedentary behavior across settings. In addition, although previous intervention research has primarily focused on reducing occupational sitting [39,44,45], the current intervention emphasized reducing sitting and promoting activity among adults across settings. Additionally, accelerometers were used to provide an objective measure of sedentary and active time; however, the accelerometers used in the study are not able to differentiate between sitting and standing. Because the mobile phone intervention messages encouraged both standing and moving around, it is noteworthy that the accelerometers used in this study did not have the capability of capturing increased time spent standing unless individuals were also moving around. Thus, it is possible that the impact of the mobile phone intervention on sedentary behavior was underestimated. It is also notable that mobile phone prompts to interrupt sedentary behavior were triggered based on self-reported sitting during random assessments throughout the day rather than objective activity monitoring.

A major limitation of the study was the quasi-experimental design (ie, nonrandom assignment). Sequential assignment of participants to the groups resulted in differences in participant characteristics including race, age, education, and smoking level. Although we attempted to control for differences in participant characteristics, randomization will be required to confirm study findings. Nevertheless, it is noteworthy that intervention participants seemed to be more vulnerable in many ways than the control group (eg, less education, more nonwhite, more smoking) and it seems promising that the intervention appeared to have a positive impact. Another limitation was that the intervention started during the first study visit (because it was embedded in the larger parent study) and, as a result, there was no baseline accelerometer measurement period. As such, we are unable to determine whether there were differences in objectively measured sedentary time between groups before the initiation of the intervention, although preintervention self-reports of daily sitting did not differ between groups. Finally, the study is limited by the short duration of the intervention (7 days). Randomized controlled trials will be needed to confirm these pilot study findings and determine the longer-term effectiveness of using mobile phone interventions to modify sedentary time.

In summary, although evidence indicates the importance of reducing and breaking up sedentary time throughout the day, it remains unclear how to most effectively reduce sedentary behavior. This study evaluated the impact of an intervention that used mobile phone technology to prompt adults to reduce and break up their sedentary time and thereby increase activity. Intervention participants had less sedentary and more active time than control participants did during the 7-day study period. Findings also suggest that simple mobile phone messages may acutely increase activity in the 10 minutes following the prompt. These findings, although preliminary, underscore the potential impact of mobile phone interventions to modify sedentary behavior and positively influence health. Effective mobile phone interventions for sedentary behavior could be a practical and wide-reaching tool for cancer and disease prevention.

## Abbreviations

BMI: body mass index
IPAQ: International Physical Activity Questionnaire
LMM: linear mixed model
REALM: Rapid Estimate of Adult Literacy in Medicine
